# Exosomal L1CAM Stimulates Glioblastoma Cell Motility, Proliferation, and Invasiveness

**DOI:** 10.3390/ijms20163982

**Published:** 2019-08-16

**Authors:** Karma R. Pace, Reetika Dutt, Deni S. Galileo

**Affiliations:** 1 Department of Biological Sciences, University of Delaware, Newark, DE 19716, USA; 2 Department of Biological Sciences, Delaware State University, Dover, DE 19901, USA; 3 Department of Chemistry and Biochemistry, University of Delaware, Newark, DE 19716, USA

**Keywords:** exosomes, glioblastoma, L1CAM, fibroblast growth factor receptor, focal adhesion kinase, cell motility, cell invasion

## Abstract

Immunoglobulin superfamily protein L1CAM (L1, CD171) normally facilitates neuronal migration, differentiation, and axon guidance during development. Many types of cancers, including glioblastoma (GBM), also abnormally express L1, and this has been associated with poor prognosis due to increased cell proliferation, invasiveness, or metastasis. We showed previously that the soluble L1 ectodomain, which is proteolyzed from the transmembrane form, can stimulate proliferation and motility of GBM cells in vitro by acting through integrins and fibroblast growth factor receptors (FGFRs). Minute L1-decorated exosomal vesicles also are released by GBM cells and potentially could stimulate cell motility, proliferation, and invasiveness, but this needed to be demonstrated. In the present study, we aimed to determine if minute L1-decorated extracellular vesicles (exosomes) were capable of stimulating GBM cell motility, proliferation, and invasiveness. L1-decorated exosomes were isolated from the conditioned media of the human T98G GBM cell line and were evaluated for their effects on the behavior of glioma cell lines and primary tumor cells. L1-decorated exosomes significantly increased cell velocity in the three human glioma cells tested (T98G/shL1, U-118 MG, and primary GBM cells) in a highly quantitative *SuperScratch* assay compared to L1-reduced exosomes from L1-attenuated T98G/shL1 cells. They also caused a marked increase in cell proliferation as determined by DNA cell cycle analysis and cell counting. In addition, L1-decorated exosomes facilitated initial GBM cell invasion when mixed with non-invasive T98G/shL1 cells in our chick embryo brain tumor model, whereas mixing with L1-reduced exosomes did not. Chemical inhibitors against focal adhesion kinase (FAK) and fibroblast growth factor receptor (FGFR) decreased L1-mediated motility and proliferation to varying degrees. These novel data show that L1-decoratred exosomes stimulate motility, proliferation and invasion to influence GBM cell behavior, which adds to the complexity of how L1 stimulates cancer cells through not only soluble ectodomain but also through exosomes.

## 1. Introduction

Tumors of the central nervous system (CNS) are a devastating disease. Of the 17,000 primary brain tumors diagnosed each year, approximately 60% are gliomas [1], which arise in the brain from the supporting glial cells or their precursors [2]. Gliomas in the brain typically are dangerous whether malignant or benign because of their location [2]. Unfortunately, symptoms often do not present in patients until the tumor progresses, leading to late detection and poor prognosis [3]. The World Health Organization (WHO) has graded gliomas from grades I-IV based on cell morphology, malignancy, and pathogenicity [2]. Low grade gliomas are characterized by cellular morphology and cause local effects that do not spread in the brain. High grade gliomas are malignant and can spread throughout the brain tissue. Glioblastoma multiforme, or simply glioblastoma, (GBM; grade IV) account for 15% of all brain tumors and are classified as such due to their extensive differentiation, high invasiveness, and thus high malignancy of the tumor [2,3,4]. The mean survival time of a patient from initial GBM diagnosis is approximately 15 months [5] due to their highly invasive nature and late diagnosis after a patient experiences headaches, seizures, memory loss, vision changes, and personality changes. Current treatments for GBM include surgery, radiation, and chemotherapy [3,5] but these are practically ineffective.

One of the factors that increases glioma cell proliferation, motility, and invasiveness is autocrine/paracrine stimulation by cell adhesion molecule L1CAM (L1, CD171). L1 normally is expressed in developing neurons and is located on the axonal membrane, but it also is expressed in gliomas [6,7]. L1 has an extracellular ectodomain with five fibronectin domains and 6 immunoglobin-like domains, which is often excised and released in extracellular fluid. L1 interacts with various binding partners including itself, cell surface integrins, and other cellular surface components [8]. L1 has a molecular weight of approximately 220 kDa and has an Arginine, Guanine and Aspartic acid (RGD) domain that binds to several integrin receptors [9]. Two L1 binding partners that are particularly important for gliomas are integrins, which activate focal adhesion kinase (FAK), and fibroblast growth factor receptor (FGFR) [10,11]. Previously, our lab and others explored the interaction between L1 with these receptors along with the source of L1 expression [11,12,13]. We have shown previously that L1-containing media and L1 ectodomain can increase migration of glioma cells [12,13,14].

L1 also is present decorating the surface of minute exosomes, which are 40–100 nm membranous vesicles, released by glioma cells and formed by inward budding of the late endosomal multivesicular body membrane [15]. Exosomes initially were isolated from blood samples and secreted by reticulocytes during differentiation [16]. Tumor samples have been found to be rich in exosomes, where they induce tumor invasion and support tumor cell survival by promoting their invasive properties [17,18,19,20,21]. We previously showed that glioma cells released exosomes decorated with L1 [12], which raised the possibility that this, too, may be a source of L1 that could autocrine/paracrine stimulate glioma cell proliferation, motility, and invasion.

In this study, we aimed to determine if minute L1-decorated extracellular vesicles (exosomes) were capable of stimulating GBM cell motility, proliferation, and invasiveness. To do this, tumor cell exosomes, including those decorated with L1, were isolated from GBM cell culture media, and the effects of L1-decorated exosomes on glioma cell migration and proliferation were explored for their stimulatory activity. We evaluated the ability of L1-decorated exosomes to stimulate glioma cell motility and proliferation in vitro and invasion into embryonic chick brain tissue in vivo. We found that L1-decorated exosomes increased the migration and proliferation rates of glioma cells. We also used specific inhibitors against FAK and FGFR to implicate specific pathways leading to stimulation. Use of nanomolar concentrations of FAK and FGFR inhibitors resulted in a reduction in the L1-decorated exosomal stimulation of migration and proliferation rates back to below untreated control levels. Finally, we explored the ability of L1-decorated exosomes to promote invasiveness of glioma cells in vivo. There was an increased initial invasion of glioma cells when the L1-decorated exosomes were mixed with L1-attenuated glioma cells before injections, but this invasion did not continue far into brain tissue, indicating a sustained need for L1 stimulation of these cells for continuous invasion.

## 2. Results

### 2.1. Exosome Characterization

**Figure 1:**
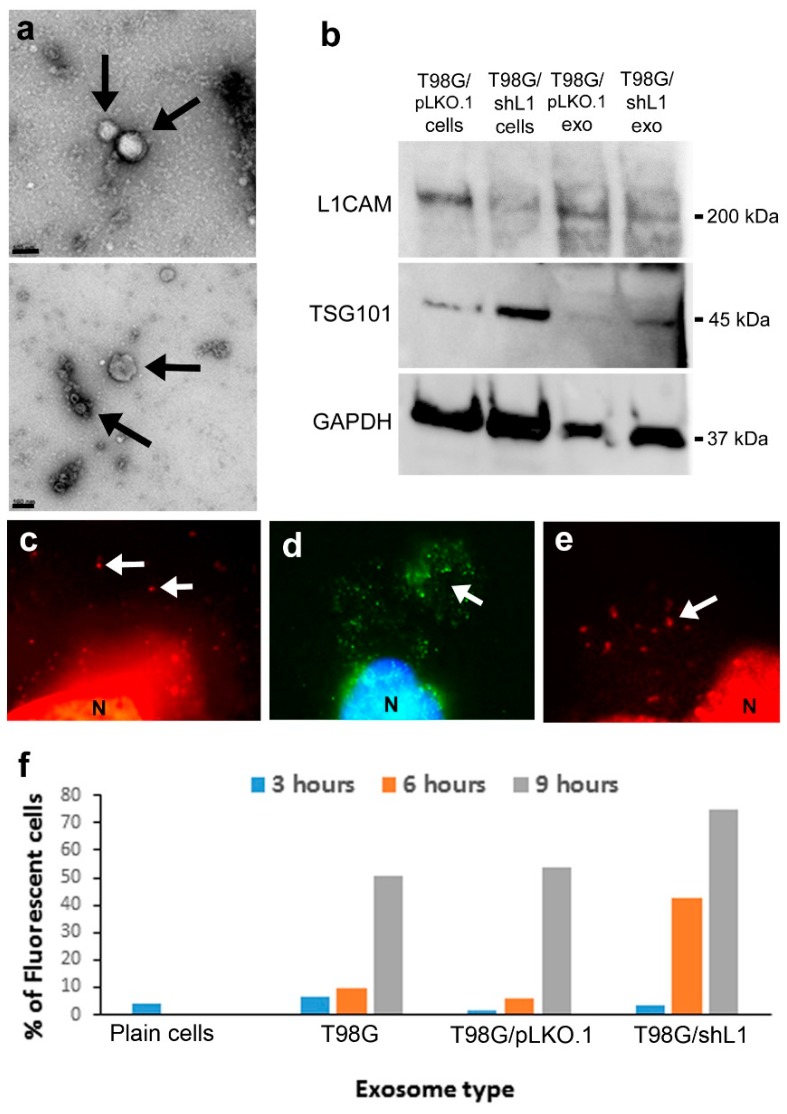
Exosome characterization and binding. (**a**) Electron micrographs of exosomes (arrows) isolated by ultracentrifugation. Scale bars = 100 nm. (**b**) Western blot analysis of cells and isolated exosome preparations. Blot was stained for L1 and then stripped and stained for TSG101 and GAPDH. (**c**) Visualization of FM 4-64 stained fluorescent exosomes binding to live cells. They appeared as small red puncta (arrows), *N* nucleus. (**d**) Exosomes stained with fluorescent Vybrant DiO resulted in bright green puncta (arrow) on cell surfaces, *N* blue nucleus stained with bisbenzimide. (**e**) Exosomes bound to cells stained for L1 with UJ127 antibody and red secondary (arrow), *N* nucleus. (**f**) DiO stained exosome uptake by T98G/shL1 cells over time. The exosomes were incubated with the cells for 3, 6, or 9 h. Cells then were analyzed for fluorescence intensity using flow cytometry. Cells showed increased fluorescence over time, and thus uptake of exosomes, by 6 or 9 h. The plain cell sample was the initial fluorescence of the cells with no exosomes added. Data in (f) are from one uptake experiment.

In this work, the term “exosomes” refers to the pellet fraction recovered after the filtration and final ultracentrifugation process described in the methods, which is an accepted method used previously by our lab and others [12,22]. The presence of exosomes isolated by ultracentrifugation were verified using the staining and imaging techniques described in the methods section. This preparation might include some other very small cellular debris such as membrane blebs or pieces, so this is a limitation of this study. However, any membrane blebs or pieces would be less than 0.2 µm and, therefore, would exclude larger microvesicles [12,22] and other larger membrane vesicles or debris. T98G, T98G/pLKO.1, and T98G/shL1 cell-conditioned low-serum media was used to isolate exosomes. The T98G cell line was chosen because those cells express L1, can be stimulated by L1, and produce exosomes [12]. Several different techniques were used to characterize exosomes isolated from T98G cell-conditioned media. One technique, transmission electron microscopy (TEM), was used to image exosomes as round vesicles between 20–100 nm in size. Figure 1 shows exosomes that have been fixed and imaged by TEM. In this preparation (Figure 1a), membrane exosomes are shown within the size range of approximately 20–100 nm. Additional exosomes were found throughout these images with varying sizes from 20–100 nm. These sizes are within known size range of exosomes [15,16].

Exosomes were analyzed by western blotting for L1 and other markers. Control T98G/pLKO.1 cells showed a prominent positive band for L1, whereas T98G/shL1 cells showed a significant reduction in L1 protein expression (Figure 1b), as shown by approximately equal GAPDH loading control staining. Correspondingly, exosomes from control T98G/pLKO.1 cells showed greater staining for L1 than did exosomes from T98G/shL1 cells, especially if taking into consideration that slightly less T98G/pLKO.1 exosomes appear to have been loaded than T98G/shL1 exosomes if normalized to either GAPDH or TSG101 bands. Exosomes from both cell types showed staining for the exosome marker TSG101 [12,22]. However, T98G/shL1 cells appeared to express more TSG101 than control cells. Exosomes from these cells showed a similar pattern, with more TSG101 in T98G/shL1 exosomes than in control exosomes. Thus, GAPDH appeared to be a better marker for normalization of exosomes than TSG101, presumably due to exosomal volume being relatively constant (along with any trapped cytoplasmic markers), whereas the relative amounts of membrane proteins may change.

Exosomes also were stained with two lipophilic membrane dyes, FM 4-64 and Vybrant DiO, which can be used to trace cellular adhesion, fusion, and migration. Stained exosomes were allowed to bind to cells on coverslips for one hour, and resulting attached exosomes were visualized as fluorescent cell surface puncta as shown in Figure 1c,d. In Figure 1c, exosomes were stained with FM 4-64, and the arrow indicates small red punctate exosomes on the cell surface (large red region on bottom of image is the nucleus). Shown in Figure 1d are exosomes stained with green Vybrant DiO, where exosomes appear as small green puncta. Cells with adherent DiO labeled T98G/pLKO.1 exosomes also were stained either for L1 (Figure 1e) or for the exosomal marker TSG101. Thus, exosomes bind to live cells within an hour, and this binding can be visualized with fluorescence microscopy.

To characterize the kinetics of exosome uptake by cells and the effects of exosomal L1 in this process, fluorescent DiO-stained exosomes were added to T98G/shL1 cell monolayers and incubated for 0 to 9 h to determine the length of time it took for exosomes to bind to the glioma cells and/or be internalized. Once the incubation periods were over, cells were lightly trypsinized and analyzed by flow cytometry for increases in fluorescence, where an increase was an indication of exosome binding and/or uptake (which these experiments cannot differentiate between). As seen in Figure 1f, cell fluorescence increased over time when incubated with labeled exosomes, indicating exosome binding and/or uptake. Average fluorescence levels of the analyzed cell populations were used to prepare the graphs. Interestingly, cells with the brightest fluorescence levels at 6- and 9-h time points were those incubated with T98G/shL1 exosomes with attenuated L1. For example, when T98G exosomes were added, there was an increase in fluorescence from 6% of cells to 51% after 9 h. The increase was greater using shL1 exosome addition to cells, with 4% to 75%. Although speculative, this may indicate that the presence of L1 on exosomes decreases their uptake kinetics, possibly by sterically blocking receptor-ligand interactions or reducing the content of other proteins that facilitate this exosome binding. Alternatively, there may be more exosomes in the T98G/shL1 preparations (as suggested by the western blot analysis above), which could bias their kinetics toward more rapid uptake. Nevertheless, exosomes of both types were bound and/or taken up by T98G/shL1 cells within 6–9 h.

### 2.2. Exosomes Increased Motility of Glioma Cells

Previously, our lab showed that conditioned media containing soluble L1 ectodomain (referred to as L1ED or L1LE) could stimulate migration of glioma cell lines in culture [12]. The migration rate for T98G/shL1 cells approached approximately 2× greater than that of cultures exposed to conditioned media lacking L1ED. Given these results, it was important to determine if L1-decorated exosomes also were capable of causing a similar increase in motility. T98G-conditioned media samples were centrifuged to separate the exosomal and supernatant fractions. These fractions were applied to the three different glioma cell lines, U-118 MG, T98G/shL1, and T98G/dFGFR to determine effects on their motility rates. As stated previously, exosomes are defined as the pellet fraction that resulted from the procedure described in the methods and characterized in the previous section.

**Figure 2:**
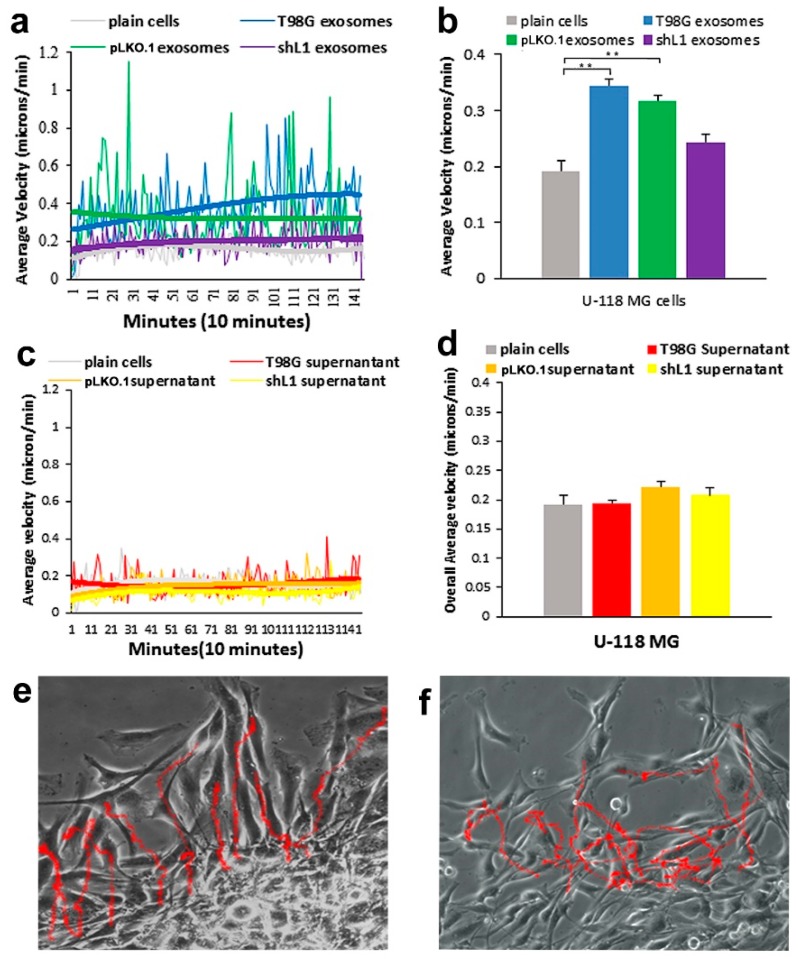
Motility of U-118 MG cells after exosome and supernatant fraction treatment. (**a**) Average velocity of cells tracked over time with exosome fractions added. The trend lines (thick lines) for T98G and pLKO.1 exosome types were distinctly above those of the plain cells and shL1 exosomes. (**b**) Overall average velocity of cells with exosome treatments. (**c**) Average velocity of cells tracked over time with supernatant fractions added. (**d**) Overall average velocity of cells with supernatant treatments. There was no significance difference in these motility rates with ANOVA analysis. (**e**,**f**) Cell track images of cells with T98G exosomes and T98G supernatant fractions, respectively. ** *p* < 0.001.

Addition of exosomes from the two T98G cells lines expressing L1 (T98G and T98G/pLKO.1) resulted in substantial and significant increases in the motility rates of U-118 MG cells in the *SuperScratch* assay, but addition of the supernatant fractions did not. For these experiments, two types of graphs are shown in Figure 2. One graph shows the average velocities of the tracked cells over time from a representative experiment (Figure 2a,c), while the other graph shows the “overall averages” in bar graph form (Figure 2b,d), which were generated from all individual cell velocity values at each time point over the entire experiment from several combined experiments. The cells increased their motility rates very rapidly after the onset of the experiments (within 10 min, Figure 2a). U-118 MG cells (L1-negative cell line), exhibited an increased motility rate of 44% overall from the addition of exosomes (Figure 2b). The greatest migration rates were found after treatment with T98G and pLKO.1 exosomal fractions. There was a significant difference between the T98G and pLKO.1 exosome migration rates when compared with plain (untreated) cells from analysis by ANOVA (p = 2.9 × 10^−7^). An example image of the U-118 cell “tracks” with added exosome fraction are shown in Figure 2e.

When supernatant fractions from conditioned media were added to U-118 cells, there essentially was no increase in migration rates, and results were similar between supernatant type. As seen in Figure 2c, the addition of supernatants did not increase the average motility rates of the cells, which can be seen by the almost completely overlapping best fit curves. The cell track images with supernatant media were similar for all the types of supernatants. In addition, when the cells were tracked, there was no significant change between the migration rates of the supernatant media and that of the untreated plain cells as seen in Figure 2d. An example image of the U-118 cell “tracks” with added supernatant fraction are shown in Figure 2f.

**Figure 3:**
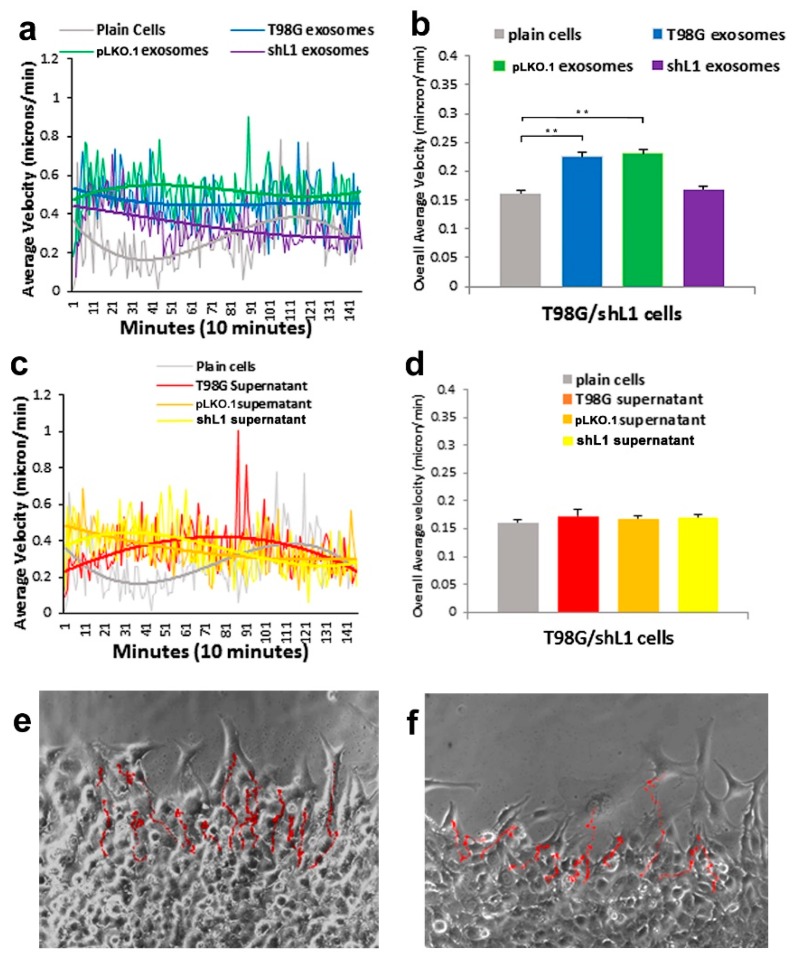
Motility of L1-attenuated T98G/shL1 cells after exosome and supernatant fraction treatment. (**a**) Average velocity of cells tracked over time with exosome fractions added. The trend lines (thick lines) for T98G and pLKO.1 exosome types were distinctly above those of the plain cells and shL1 exosomes. (**b**) Overall average velocity of cells with exosome treatments. (**c**) Average velocity of cells tracked over time with supernatant fractions added. (**d**) Overall average velocity of cells with supernatant treatments. There was no significance difference in these motility rates with ANOVA analysis. (**e**,**f**) Cell track images of cells with T98G exosomes and T98G supernatant fractions, respectively. ** *p* < 0.001.

L1-attenuated T98G/shL1 cell motility also was tracked after treatments with exosome and supernatant fractions. As shown in Figure 3a,b, cells were stimulated by the T98G and T98G/pLKO.1 exosomal fractions, but not by T98G/shL1 exosomal fractions. The overall average migration of T98G/shL1 glioma cells showed a significant increase (*p* < 0.001) in velocity rates with the T98G and pLKO.1 exosomes (27% and 30%, respectively; Figure 3b). An example image of T98G/shL1 cell “tracks” with added exosome fraction are shown in Figure 3e. As seen for U-118 MG cells, when the different supernatant fractions were added to the T98G/shL1 cells, there was no increase in motility rates by inspecting best fit curves with added supernatant fractions (Figure 3c). Overall average motility graphs (Figure 3d) showed no statistical difference between the motility rates of the supernatant fractions. An example image of the T98G/shL1 cell “tracks” with an added supernatant fraction is shown in Figure 3f.

**Figure 4:**
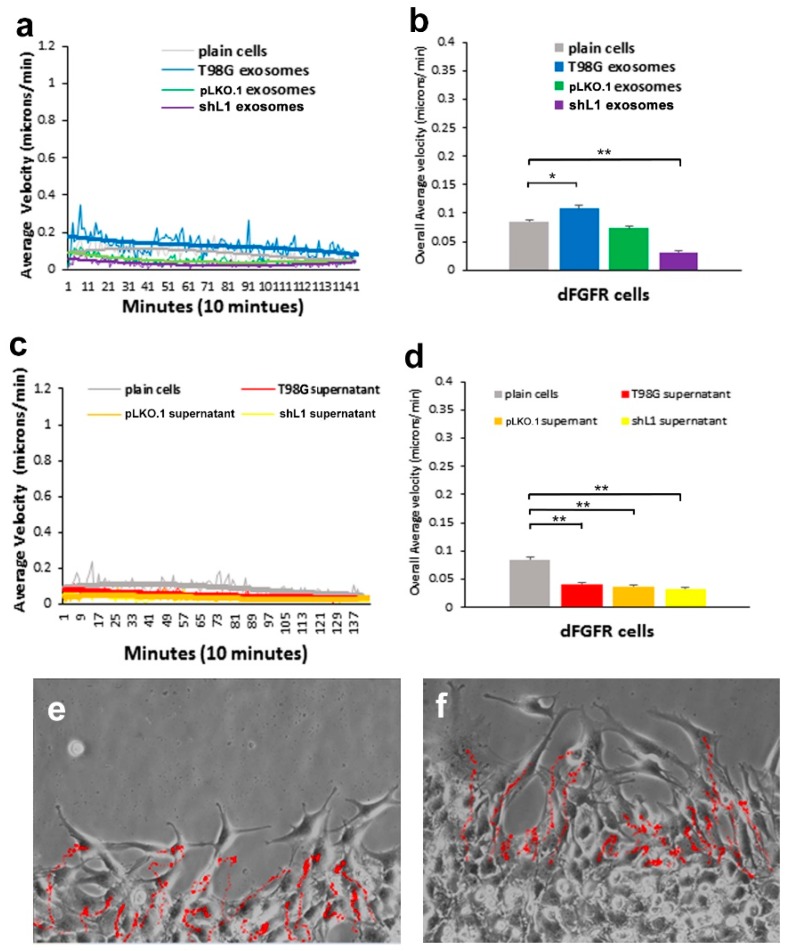
Motility of T98G/dFGFR cells after exosome and supernatant fraction treatment. (**a**) Average velocity of cells tracked over time with exosome fractions added. The trend line (thick line) for T98G exosome type was distinctly above those of the plain cells and shL1 exosomes. (**b**) Overall average velocity of cells with exosome treatments. (**c**) Average velocity of cells tracked over time with supernatant fractions added. (**d**) Overall average velocity of cells with supernatant treatments. Motility rates of treated cells were significantly lower than untreated. (**e**,**f**) Cell track images of cells with T98G exosomes and T98G supernatant fractions, respectively. * *p* < 0.05, ** *p* < 0.01.

To compare the effects of L1-decorated exosomes on glioma cells with decreased FGFR activity, T98G/dFGFR glioma cells made and characterized previously [13] were used in the motility analysis. As these cells overexpress a dominant-negative kinase-deficient FGFR1 receptor, their motility was even less than T98G/shL1 cells. As shown in Figure 4a,b, only the T98G exosome fraction increased motility of dFGFR glioma cells (27%, Figure 4b) when compared to untreated plain cells. This increase was found to be significant based on analysis using ANOVA (p = 2.5 × 10^−20^). With supernatant fraction treatment, there was significantly lowered motility using all the different supernatants from various glioma cells (Figure 4c,d). When compared to the plain T98G/dFGFR cells, these supernatants showed significantly less motility for unknown reasons. Example images of the T98G/dFGFR cell “tracks” are shown in Figure 4e,f. These results taken together show that L1-decorated exosomes significantly increased motility of the glioma cell lines tested when applied, but supernatant fractions did not. This information is important because it further defines the exosomal fraction as a portion of GBM cell media that is capable of increasing the motility of those cells in an autocrine/paracrine manner.

In addition, we looked at the ability of L1-decorated exosomes to stimulate the motility of a previously described patient-derived GBM surgical specimen. L1-negative GBM cells (GBM #22 in [23]), which neither express nor release L1 ectodomain or L1-decorated exosomes into their culture media, showed a 57% increase in motility when treated with T98G exosomes vs. T98G/shL1 exosomes (0.144 ± 0.011 s.e.m. microns/min vs. 0.092 ± 0.010 s.e.m. microns/min). This shows that stimulation of motility by L1-decorated exosomes is not confined only to established GBM cell lines, but also operates in more recent patient-derived specimen cells.

In summary, as seen in Table 1, the L1-decorated exosomes increased the velocity rates of all glioma lines tested. However, there was no significant change in velocity rates when supernatant fractions were added to the cells. This indicates that L1-decorated exosomes in the conditioned media is a significant factor for stimulating the motility of glioma cells.

### 2.3. Exosomes Increased the Proliferation of Glioma Cells

**Figure 5:**
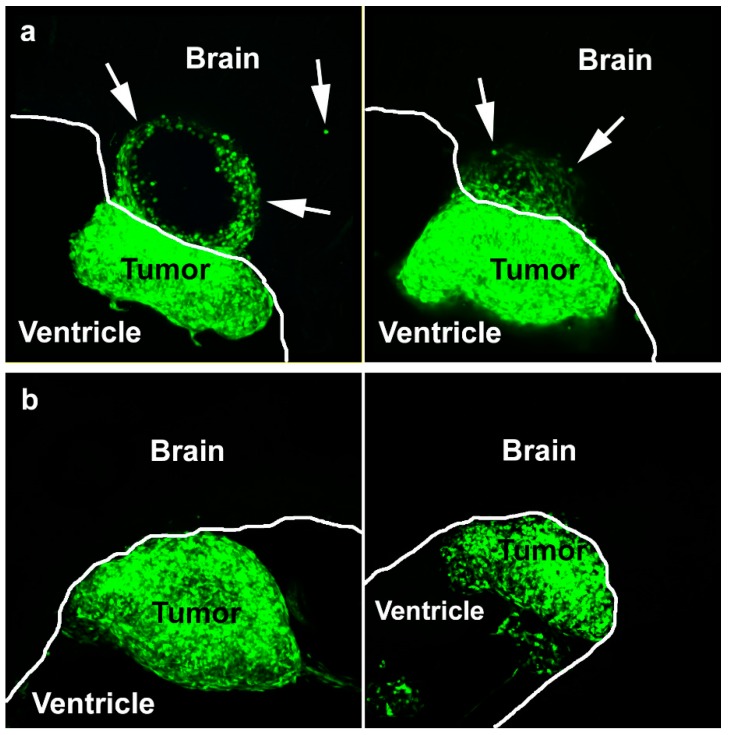
L1-decorated exosomes initiate brain invasion. (**a**) Two examples of invasion of GFP-expressing T98/shL1 cells beyond the tumor margin (arrows) when mixed with T98G L1-decorated exosomes. (**b**) Two examples of GFP expressing T98G/shL1 tumors where cells did not invade the brain beyond the tumor margin when mixed with L1-reduced T98G/shL1 exosomes. White lines show the surface of the brain wall along the ventricle. Brain tissue is unstained. Images are confocal maximum projections of z-stacks.

Another important feature of gliomas is that they are highly proliferative [6]. Increased production of glioma cells can be monitored by their advancement through the cell cycle. Due to this highly proliferative nature, glioma cells were analyzed to determine the effects of the L1-decorated exosomes on the progression through the cell cycle. Previously, we showed that L1-containing media induced glioma cell proliferation [12]. To determine the effect of L1-decorated exosomes on glioma cell proliferation, glioma cell lines were treated with T98G exosome fractions for one day, fixed, stained with propidium iodide (PI), and analyzed using flow cytometry to determine relative DNA content. The DNA content profile was converted into the percentages of each phase of the cell cycle by using ModFit LT software. An increase in the percentage of cells in S phase indicates increased proliferation among the population. The results shown in Figure 5 are from individual experiments analyzing approximately 10,000–50,000 cells. To verify proliferation by an independent method, cell counts also were performed as described in the Methods section.

In summary, all three glioblastoma cell types (U-118 MG, T98G/shL1, T98G/dFGFR) exhibited increased proliferation when treated with T98G exosomes, indicating that exosomal L1 induced progression through the cell cycle. We report here absolute percentage point increases in S phase instead of relative percentage increases, which would be even larger. Proliferation results are summarized in Table 2. There was 12.3 percentage point increase in S phase population of the U-118 MG cells after T98G exosome treatment compared to unstimulated plain cells. In addition, a cell count was performed over 2 days on the same cells to independently verify that increased cell division occurred in exosome treated cells. There was a 2-fold increase in cell number when L1-decorated exosomes were added to the U-118 MG cells when compared with the untreated plain cells. There was a 9.2 percentage point increase in S phase population of the T98G/shL1 cells after T98G exosome treatment compared to unstimulated plain cells. There was a 1.5× increase in cell number with exosome treatment compared with the untreated plain cells. Similar effects on proliferation also were seen in the T98G/dFGFR cells, where L1-decorated exosomes resulted in a 9.1 percentage point increase in the S phase population.

### 2.4. Exosomes Facilitated Glioma Cell Invasion into Brain

We showed previously that L1 expression in T98G cells facilitated invasion in our chick embryo brain tumor model system by demonstrating that L1-attenuated T98G/shL1/eGFP cells did not invade [12]. We wanted to determine if isolated L1-decorated exosomes could restore T98G cell invasion in this model system and, if so, to what extent. To determine this, T98G/shL1/eGFP cells or T98G/pLKO.1/eGFP cells were mixed with the different exosome types and injected into developing chick optic tecta on E5. These cells expressed eGFP so that they could be visualized in thick brain tissue sections [12].

The results of the in vivo experiments are presented in Table 3. The unaltered positive control T98G/eGFP cell line that normally expresses L1 and releases L1-decorated exosomes formed tumors and invaded the optic tectum as before [12]. When additional exosomes were added to those cells, tumors formed and invasion occurred, as expected. With the L1-attenuated T98G/shL1 cell line, tumors formed but exhibited a smooth tumor margin, and there was no invasion into the wall of the brain beyond that margin, as reported previously [12]. When mixed with L1-decorated exosomes, T98G/shL1/eGFP cells formed tumors with a more jagged margin, and cells migrated short distances beyond the tumor margin into the brain tissue. This indicated that L1-decorated exosomes facilitated some initial invasion in this in vivo system. This invasion of T98G/shL1 cells induced by L1-decorated exosomes was dramatically less than the cell invasion of unaltered T98G cells [12], which continuously produce L1-decorated exosomes and released L1 ectodomain to sustain cell invasiveness. T98G/shL1 cells, on the other hand, have highly attenuated L1 expression, so they presumably would be exposed to L1-decorated exosomes only during the initial injection period and soon thereafter, driving initial invasion but not sustained invasion. Cells during the remainder of the 4-day experimental period would be without a source of L1 to promote their continued invasion. Examples of invasion of T98G/ shL1/eGFP cells mixed with L1-decorated or L1-attenuated exosomes are shown in Figure 5.

### 2.5. FGFR and FAK Inhibitors Decreased Exosomal Motility and Proliferation

To determine if intracellular signaling initiated by L1-decorated exosomes was similar to that of the L1 ectodomain, fibroblast growth factor receptor (FGFR) and focal adhesion kinase (FAK) inhibitors were used. FGFRs can initiate significantly increased glioma cell motility and proliferation in response to the soluble L1 ectodomain [13,14,15]. To investigate the effects of loss of FGFR function on stimulation by L1-decorated exosomes, two strategies were used. The first strategy was to use T98G/dFGFR cells with diminished FGFR activity. The results of using this cell line in motility and proliferation studies are described above. Second, we used an FGFR kinase inhibitor (PD173074) to decrease signaling. Previously, our lab showed that use of this inhibitor at 50 nM significantly reduced the velocity of T98G cells in response to L1 ectodomain [13,14].

**Figure 6:**
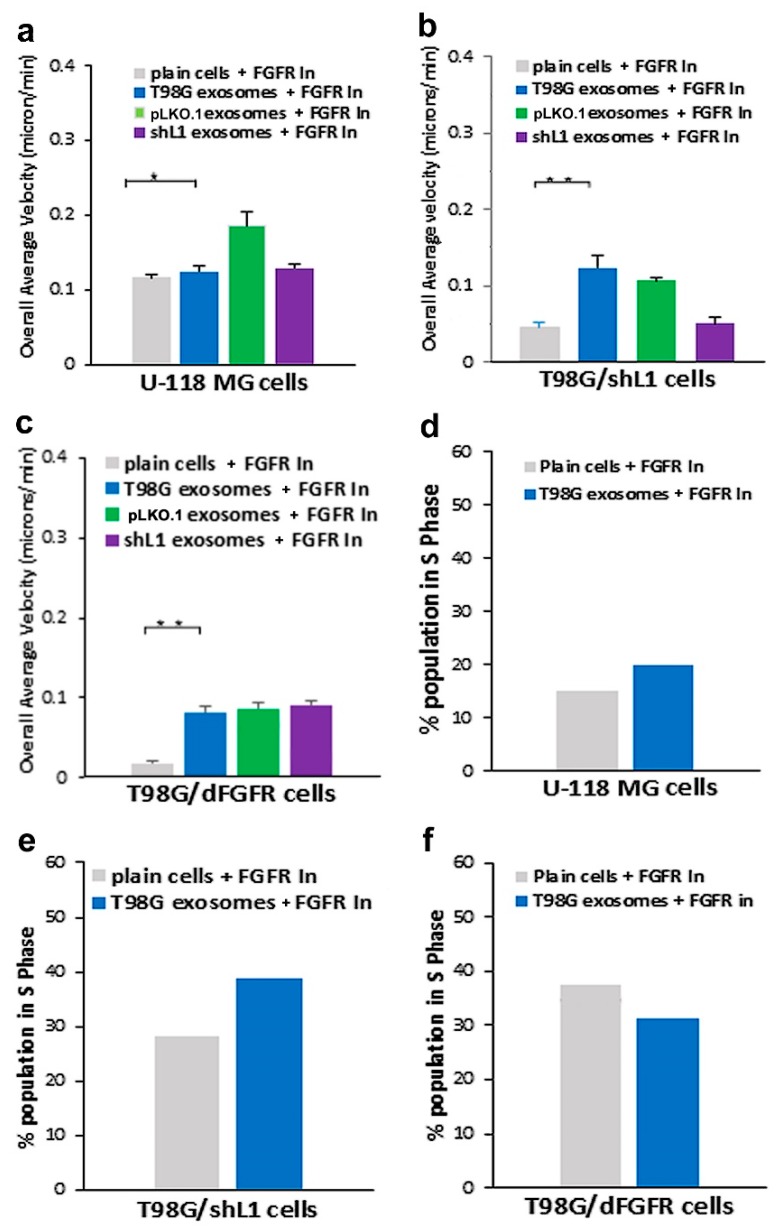
Effects of FGFR inhibitor PD173074 on motility and proliferation. (**a**) Overall average velocities of U-118 MG cells treated with the FGFR inhibitor. (**b**) Overall average velocities of T98G/shL1 cells treated with inhibitor. (**c**) Overall average velocities of T98G/dFGFR cells treated with inhibitor. (**d**) U-118 MG cell proliferation (% S phase population) with the addition of the inhibitor. (**e**) T98G/shL1 cell S phase population when treated with inhibitor. (**f**) T98G/dFGFR cell S phase population when treated with inhibitor, * *p* < 0.05, ** *p* < 0.01.

The addition of 50 nM PD173074 to U-118 MG cells resulted in decreased migration under all conditions (Figure 6a, solid bars) and almost entirely inhibited the stimulation by L1-decorated exosomes (compare to Figure 2). Thus, exosomal stimulation of U-118 cell motility was abrogated with the FGFR inhibitor. When PD173074 was added to T98G/shL1 cells, there again was a significant decrease in velocity rates (Figure 6b; compare to Figure 3). The addition of PD173074 to T98G/dFGFR cells also resulted in decreased motility of T98G exosome stimulated cells (Figure 6c; compare to Figure 4). Taken together, these results indicate that there was signifcant reduction in the ability of cells to respond to the L1-decorated exosomes due to inhibition of FGFR signaling.

When evaluting the effect of PD173074 on proliferation, addition of the inhibitor to cell cultures resulted in fewer cells in S phase when compared to the initial stimulation rates by exosomes (Figure 6d). The FGFR inhibitor had similar effects on the T98G/shL1 cells (Figure 6e). The inhibitor reduced the proliferation of both plain and exosome-stimulated cells, and the proliferation of the stimulated cells was reduced to the level of unstimulated cells without inhibitor. The additon of FGFR inhibitor showed a similar trend when added to the T98G/dFGFR cells (Figure 6f). Thus, the addition of FGFR inhibitor greatly reduced glioma cell motility and proliferation regardless of exosomal stimulation rates and, in almost all cases, abrogated the stimulatory effects of the L1-decorated exosomes.

**Figure 7:**
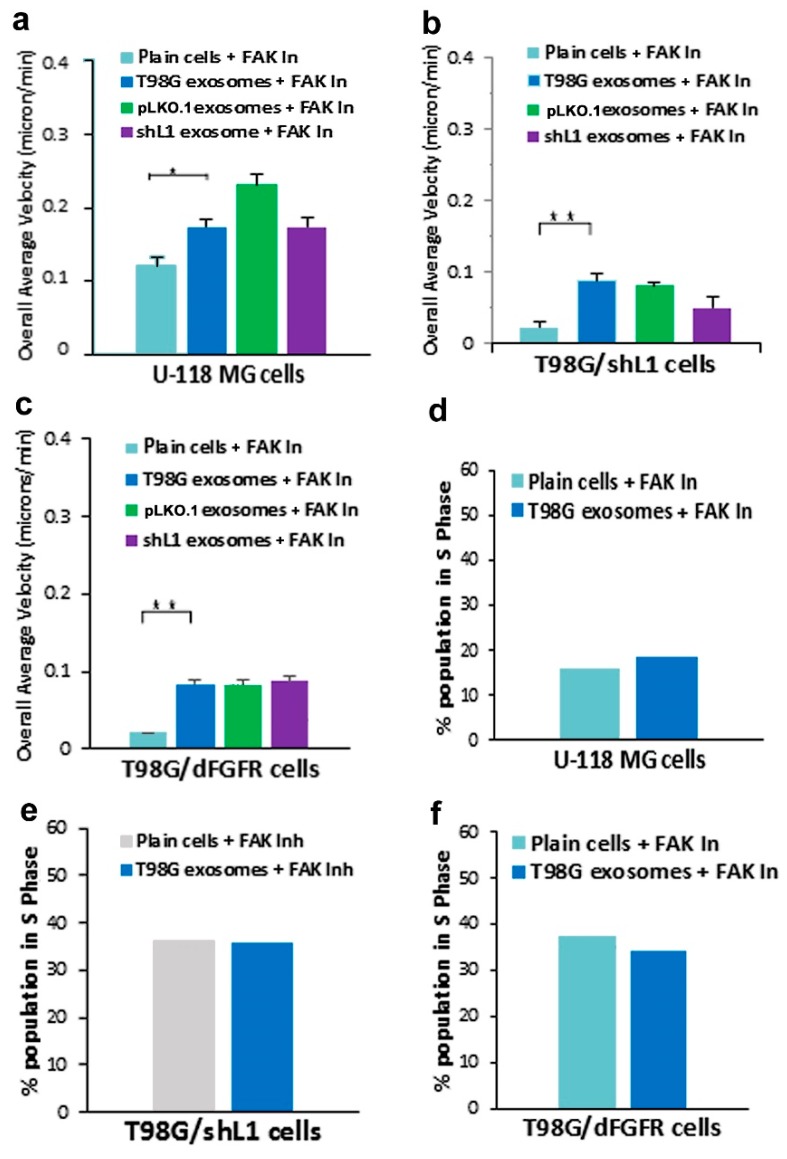
Effects of FAK inhibitor PF 431396 on motility and proliferation. (**a**) Overall average velocities of U-118 MG cells treated with the FAK inhibitor. (**b**) Overall average velocities of T98G/shL1 cells treated with inhibitor. (**c**) Overall average velocities of T98G/dFGFR cells treated with inhibitor. (**d**) U-118 MG cells had decreased proliferation (% S phase population) with the addition of the inhibitor. (**e**) T98G/shL1 cells had decreased proliferation when compared to the original stimulation. (**f**) T98G/dFGFR cells also showed a reduction in proliferation when compared to the original stimulation, * *p* < 0.05, ** *p* < 0.01.

The potential involvement of the integrin/FAK pathway during L1–decorated exosome stimulation also was studied because L1 has been shown to initiate signaling through integrins and FAK [12,14,19,20]. A FAK inhibitor (PF 431396) was used at 10 nM as before [14] to determine its effects on the motility and proliferation of glioma cells stimulated by exosomal L1. Treatment of U-118 MG cells with PF 431396 resulted in a complete abrogation of the stimulation by exosomes decorated with L1 (Figure 7a, blue solid bar; compare to Figure 2). The effect of PF 431396 also significantly reduced the increases in motility of T98G/shL1 cells treated with exosomes, with all three exosome types showing similar results (Figure 7b; compare to Figure 3). This indicates that the integrin/FAK pathway is important for L1-decorated exosome stimulation of these two cell types. FAK involvement also was investigated in the motility of T98G/dFGFR cells, which exhibited minimal stimulation by L1-decorated exosomes (Figure 7c; compare to Figure 4). The FAK inhibitor appeared to have no inhibitory effect on cells treated with exosomes, potentially indicating that signaling upstream of FAK was through FGFRs, which was already abrogated by the dominant-negative FGFR expression in these cells. This is consistent with our previous results in T98G cells where inhibition of L1 stimulation was not additive using chemical inhibitors of FGFR and FAK together [14].

In addition to these motility results, FAK inhibitor was used to determine effects on cell proliferation. FAK inhibitor reduced exosomal stimulation of S phase by approximately half in U-118 MG cells (Figure 7d). Similar results were seen with T98G/shL1 and T98G/dFGFR cells, with FAK inhibitor treatment resulting in the abrogation of L1-decorated exosomal stimulation of proliferation (Figure 7e,f). These results taken together indicate that FAK is a critical signaling component that mediates L1-decorated exosomal stimulation of motility and proliferation.

Since the individual inhibitors each reduced L1-decorated exosomal stimulation of migration and proliferation, potential additive effects of combined inhibitors were explored, as we explored for stimulation by soluble L1 ectodomain [14]. This would determine if the integrin/FAK and FGFR pathways were distinct, or if they shared downstream components. Inhibition of both pathways should reduce migration and proliferation more than either single inhibitor, unless these two pathways shared common effectors.

**Figure 8:**
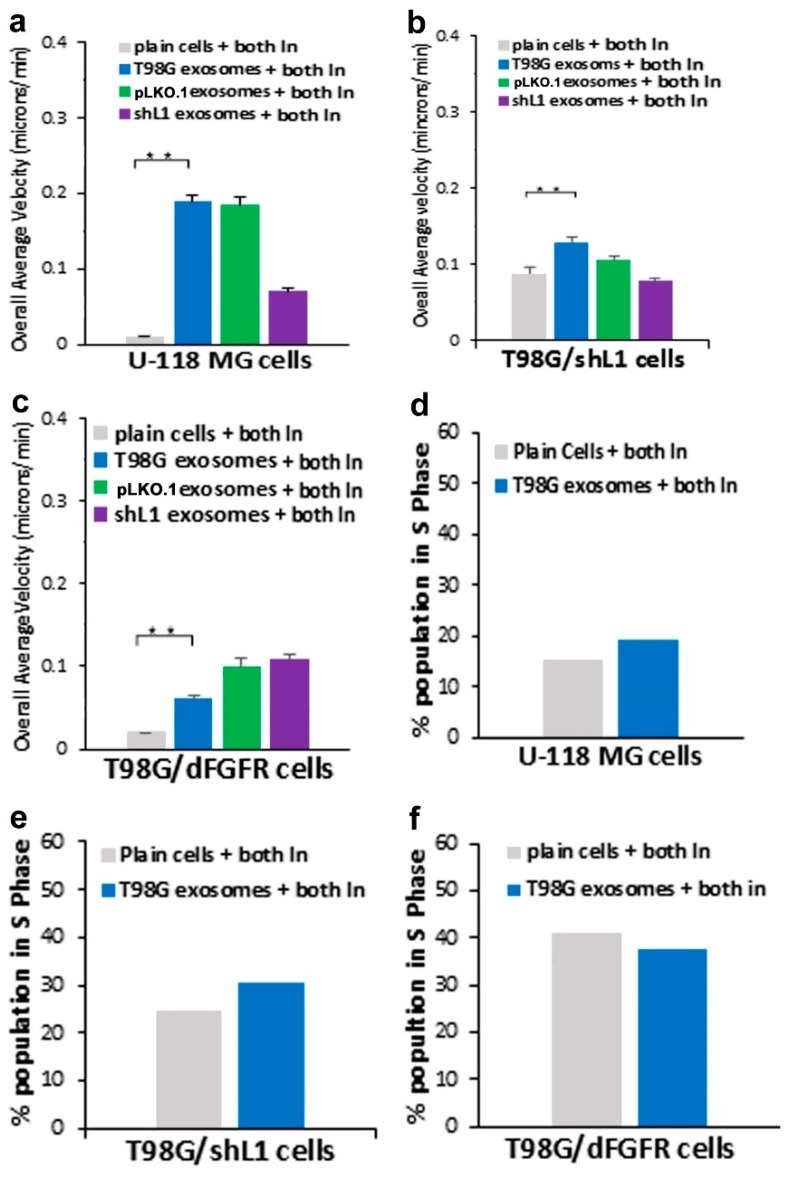
Effects of FGFR plus FAK inhibitors on motility and proliferation. (**a**) Overall average velocities of U-118 MG cells treated with both inhibitors, showing significant reduction in the migration rates with the inhibitors. (**b**) Overall average velocities of T98G/shL1 cells treated with both inhibitors. (**c**) Overall average velocities of T98G/dFGFR cells treated with both inhibitors. (**d**) U-118 MG cells had decreased proliferation (% S phase population) with the addition of both inhibitors. (**e**) T98G/shL1 cells treated with both inhibitors. (**f**) T98G/dFGFR cells treated with both inhibitors, ** *p* < 0.001.

For U-118 MG cells, stimulation by L1-decorated exosomes was reduced approximately the same as with either single inhibitor (Figure 8a). shL1-exosome stimulated U-118 cells were reduced more with both inhibitors than with single inhibitors (purple bar). For T98G/shL1 cells, none of the cells treated with both inhibitors showed lower motility than with the single lowest single inhibitor (Figure 8b). This also was true for T98G/dFGFR cells (Figure 8c), with the exception that cells stimulated with T98G exosomes were slightly slower than cells with single inhibitors, but the reductions were not additive. Thus, these inhibitors did not result in additive effects on L1-decorated exosomal mediated increases in motility.

For U-118 MG cells, the effects of combined inhibitors were not additive compared to single inhibitors (Figure 8d). For T98G/shL1 cells, treatment with combined inhibitors resulted in partially, but not completely, additive reductions in proliferation compared to single treatments (Figure 8e). For T98G/dFGFR cells, the effects of combined inhibitors were not additive compared to single inhibitors (Figure 8f).

Thus, proliferation results generally followed those of motility. The degree of additive effects was cell-type specific, with only T98G/shL1 cells showing partially additive effects. These results taken together indicate that integrin/FAK and FGFR pathways might share a common downstream component, possibly FAK itself, which appeared to be the case also for L1 ectodomain stimulation [14].

## 3. Discussion

Glioblastoma (GBM) remains practically incurable despite multi-mode treatments that are given to patients (e.g., surgical resection, chemotherapy, radiation therapy) [1,2,3,4,5]. This resistance to treatment is at least partly due to the high degree of motility and invasiveness of single cancer cells, which invade the brain tissue beyond the visible margins of the tumor. We previously identified the expression and release of L1 ectodomain as a potent autocrine/paracrine stimulator of motility, proliferation, and invasion for GBM cells [12,13,14]. Here, that work was extended to show for the first time that L1-decorated exosomal vesicles released by GBM cells also are potent stimulators of motility and proliferation in vitro and invasion in vivo. When added to cultures, L1-decorated exosomes increased migration rates of U-118 MG, T98G, and T98G/dFGFR cells. In addition, L1-decorated exosomes also increased their proliferation, as indicated by both increased S phase and cell counts. To determine the extent to which exosomes initiated stimulatory signals through FGFRs and FAK, two potent small molecule inhibitors were used. The addition of FGFR and FAK inhibitors reduced the motility increase caused by exosomal addition back to basal levels. The chick brain invasion experiments showed that L1-decorated exosomes were capable of facilitating tissue invasion by GBM cells, thus demonstrating their importance in vivo. These results taken together demonstrate the importance of exosomal L1 for stimulation of GBM cells and the effectiveness of small molecule inhibitors in abrogating that stimulation.

Our previous results have shown that migration rates of GBM cells were increased by either when L1 ectodomain-containing media was added to L1 deficient cells [12], or when L1 ectodomain was expressed by lentiviral transduction [13]. The work presented here highlights the ability of L1-decorated exosomes from GBM cells to induce a significant increase in GBM cell migration and proliferation. Thus, these two different forms of L1 (soluble ectodomain and L1-decorated exosomes) are capable of modulating the behavior of GBM cell motility, proliferation, and invasiveness. Although we showed exosomal markers and TEM images, one limitation of our study is that we have not demonstrated exclusion of non-exosomal membranes from our preparations. However, our methods would have excluded membrane vesicles larger than 0.2 microns, such as microvesicles, and other large membrane debris.

There have been previous studies that have looked at the roles of GBM exosomes, but these did not report that a single membrane protein could significantly alter cell behavior. In one study, researchers looked at the effect on migration using exosomes from U-87 MG cells, whereby the exosomes increased the migration of GBM cells when added to trans-well cultures [24]. This increased migration might have been due to L1 decorating the exosomes, since we previously showed that U-87 MG cells release L1-decorated exosomes [12]. In another relevant study [25], Skog et al. found that addition of primary GBM tumor exosomes nearly doubled the proliferation of U-87 glioma cells in vitro, but they did not ascribe this increase to any particular mechanism or protein. Those tumor exosomes were found to have migration-promoting mRNAs, as well as other factors that led to additional migration of the glioma cells [25]. Those results are similar to our increased proliferation results, which largely were due to L1.

Our lab demonstrated that the FGFR is involved in increased proliferation of GBM cells with L1 ectodomain stimulation [13,14]. Other researchers have suggested that the epidermal growth factor receptor (EGFR) expressed on the surface of exosomes may also contribute to glioma cell proliferation. Several studies reported EM images of their exosomes and have found that these exosomes contain mRNAs, protein receptors, DNA, and microRNAs [26]. There have been attempts to use exosomes as biomarkers to determine the presence of disease and other cellular dysfunction [27]. The EM images provided here are similar to those in other published work. Electron micrographs, however, lack information beyond exosomal size and shape, so several techniques have been used to better determine their structure and interaction with GBM cells. One study used atomic force microscopy to visualize the surface of a cell and the attached exosomes while preserving the 3-D structure [26].

In addition to visualization of exosomes, some studies have investigated their uptake. In this work, we showed that the uptake of fluorescent L1-decorated vs. L1-deficient exosomes differed over a 9-h time course. Binding and uptake of exosomes was clearly evident by the increasing fluorescence of glioma cells over time. These results align with other work that has shown in vitro exosome uptake [24]. Previous work also has shown that exosomes express heparin sulfate proteoglycans (HSPGs) [24], which facilitate binding to cells, and that when HSPGs were inhibited, there was a reduction in exosome binding and uptake. Our results are interesting in that L1 appeared to decrease exosome binding and uptake, or at least slow it down, although these results are not definitive and may be the result of adding different amounts of exosomes (see Results). Others also have shown that exosomes can alter the gene expression of cells that take them up. Thus, our results potentially contribute to the repertoire of molecules that modulate uptake of exosomes, which influences the behavior of GBM cells in multiple ways.

To further investigate exosomal cellular signaling, two known signaling pathways were explored. The FGFR is a known L1 receptor [28] that has been a target of glioma treatments since it was found to induce GBM when it interacts with Transforming Acidic Coiled Coil genes (TACC) [29]. Our work focused on the exosomal L1-FGFR signaling. Previously, we showed that L1 stimulates motility and proliferation of GBM cells in culture via FGFRs [13]. The cell line with dominant-negative FGFR (T98G/dFGFR) showed decreased migration rates with soluble L1 ectodomain-conditioned media stimulation as compared to cells with normal FGFRs. This study extended that work to investigate the effect of L1-decorated exosomes on motility and proliferation via FGFRs. To reduce FGFR signaling, a potent inhibitor of FGFR (PD 173074) was used. When applied to cells in a *SuperScratch* assay [30], this FGFR inhibitor resulted in a marked reduction of the ability of exosomes to stimulate glioma motility. These motility rates went below those of plain T98G/shL1 cells, which indicates that other FGFR ligands (e.g., bFGF) may stimulate motility of T98G cells. Attenuation of FGFR signaling via the dominant-negative approach (T98G/dFGFR cells) resulted in the lowest motility rates seen in these studies. These findings support the idea that L1-decorated exosomes initiate sufficient signaling via the FGFR pathway to stimulate motility.

Another kinase explored in this work was FAK, which was presumed to become activated as a result of L1 stimulation of integrins. To explore the effect of L1-decorated exosomes on FAK signaling, a FAK inhibitor (PF 431396) was added to gliomas in a *SuperScratch* assay. This FAK inhibitor significantly decreased the migration rate of glioma cell lines when added with exosomes. U-118 MG cell motility was reduced by about half compared to those stimulated with L1-decorated exosomes. The T98G/shL1 and T98G/dFGF cells showed significant reduction in velocity as well. L1-decorated exosomal stimulation of proliferation also was significantly reduced. This correlates with the published research findings that FAK inhibition reduces glioma progression and that FAK inhibition reduced tumor size of GBM, thus making this a viable clinical treatment for GBM [31].

Although it was hypothesized by us that additional significant inhibition (e.g., additive effects) might be seen with the combination of both inhibitors, the results did not show much difference from either inhibitor alone. The addition of both inhibitors showed similar motility results to FGFR and FAK inhibitors individually for all GBM cell types used. This suggests that maximum motility inhibition had occurred because they share a common pathway, possibly FAK itself, as hypothesized by us previously for stimulation by L1 ectodomain [14].

Our proliferation data showed that the addition of L1-decorated exosomes significantly increased cell cycle progression (the percentage of cells in S phase) of GBM cells. This is relevant to other studies that have investigated the effects of exosomes on proliferation. In one study, exosomes were isolated from gastric cancer cells and shown to promote proliferation through the PI3K/Akt and MAPK/ERK activation [32]. In another study, exosomes from MDA-MB-231 cells were shown to promote proliferation of umbilical cord endothelial cells [33]. In addition, exosomes from chronic myeloid leukemia and nasopharyngeal carcinoma tumors were used to promote proliferation of lymphocytes and other cells by increasing BCL-w, BCL-xl, and survivin, and a reduction of the pro-apoptotic molecules BAD, BAX and PUMA [34]. Our results showing that L1-decorated exosomes promoted proliferation add another mechanism by which exosomes stimulate proliferation.

Based on the in vitro data that L1-decorated exosomes increased GBM cell motility, T98G/shL1 cells mixed with exosomes were injected into the optic tectum of developing chick embryos. Previously, it was shown that human and rat glioma cell lines could develop invasive tumors when injected into chick tectum [35] and that L1 was necessary for invasion by T98G cells [12]. Another in vivo study by others implicated L1 in GBM cell invasion by a correlative pattern of L1 immunostaining surrounding cells at the invasive front [36]. In our work here, it is reported that L1-deficient T98G cells can form invasive tumors in chick brains only when mixed with L1-decorated exosomes, and not with L1-deficient exosomes. With L1-decorated exosomes, there was invasion into the optic tectum tissue beyond the generally smooth tumor margin, however, it was not as extensive as with normal T98G cells [12]. We interpret this to reflect an initial stimulation by the exosomes, which was not sustained as the cells invaded into the tissue without further exogenous exosomal L1 stimulation. These data add to the idea that L1, as a component of exosomes, may be an important factor for GBM cell invasion in vivo. Therefore, L1 is a potential target for treatment, since patients that receive surgery have high recurrence of GBM. Since FAK and FGFR inhibitors used here were effective against L1-decorated exosomal stimulation, and they also were effective against L1 ectodomain stimulation [14], they may be useful as treatments for GBM tumor cells that express, and are stimulated by, L1. However, our work here using two GBM cell lines (T98G and U-118 MG) shows that GBM cells that share the common characteristic of L1 stimulation may respond differently to such inhibitors.

## 4. Materials and Methods

### 4.1. Cell Lines and Cultures

Several cell lines and primary tissues were used for this work. For many experiments, two glioma cell lines, T98G and U-118 MG, were used. Both of these cell lines were obtained from the American Type Culture Collection (ATCC, Manassas, VA). T98G, a human WHO Grade IV glioma cell line [37], expresses endogenous L1 [12]. Another cell line was created (T98G/shL1) with a lentiviral vector insertion of a short hairpin RNA in T98G to reduce L1 expression (Open Biosystems TCTN0000063917; RHS3979-97052304) [12]. The non-targeting control vector pLKO.1 with puromycin selection was used to create control T98G/pLKO.1 cell line. T98G/dFGFR cells [13] are T98G cells that overexpress a dominant-negative FGFR1 construct that abrogates FGFR activity. U-118 MG, a human WHO Grade IV glioma line, does not express endogenous L1 protein [12]. These two cell lines were used in comparison to the T98G lines to indicate endogenous vs exogenous L1 function. In addition, two surgical GBM samples of patient primary tumor cells from the Tissue Procurement Center of The Helen F. Graham Cancer Center and Research Institute of Christiana Hospital (Newark, DE) also were used in same experiments to compare cell lines to primary tumor sample to evaluate similarity to cultured lines. All tissues were cultured in Dulbecco’s Minimal Eagle’s Medium (DMEM) supplemented with 10% Fetal Bovine Serum (FBS), 2 mM Penicillin/ Streptomycin (Pen/Strep) and 1 mM L-Glutamine (L-Glut). The tissues were incubated at 37° C with 5% CO_2_.

### 4.2. Exosome Collection

Exosomes were isolated from T98G cell line culture media using ultracentrifugation. Cells were plated until 75% confluent in complete culture media (10% FBS). The complete media was removed from the plates and the cells were rinsed with Phosphate Buffer Saline (PBS). Next, DMEM containing 1% FBS, 2 mM Pen/Strep and L-Glut (low serum media), was added to cultured cells which were grown to 80–90% confluency for 24 h. This conditioned media was collected, centrifuged at low force (800 rpm) to remove any cells, and then supernatant was filtered using a 0.2 µm filters to remove any remaining cellular debris. This filtered media then was centrifuged at high speed for separation of exosomes from supernatant. The filtered media was centrifuged for 10 min at 1200× g and collecting supernatant, then 20 min at 10,000× g collecting supernatant, and finally 22 h at 100,000× g in an ultracentrifuge using a swinging bucket rotor. After this final centrifugation, the supernatant fraction (devoid of exosomes) and pellet fraction (containing exosomes) were used in experiments including motility and proliferation. These exosome pellets were gently rinsed with PBS, suspended in low serum (0.5–1% FBS) media, and added to cultured cells as needed. Typically, the exosomes isolated from a 10 cm culture were resuspended in 30–50 µL of low serum media. Some exosomes were used immediately, while others were stored for up to 30 days at 4 °C for later use.

### 4.3. SDS PAGE and Western Blotting

Cells on culture dishes were rinsed with phosphate buffered saline (PBS) and then solubilized in RIPA lysis buffer with Complete Mini Protease Inhibitor (Roche) for 3–5 min on ice. Cells were scraped, lysed by sonication and centrifuged to collect the cell lysates. Protein quantification was performed using the Bradford Assay (Sigma). Exosome pellets were dissolved in 100 μL RIPA lysis buffer with protease inhibitor for use in western blotting. 20 μg of protein from cell lysate and 50μL of exosome fraction were used to load lanes on the gel. Samples were prepared using NuPAGE LDS Sample Buffer (NP0007) and NuPAGE Sample Reducing Agent (NP0009) and were electrophoresed on Novex WedgeWell 4–20% Tris-Glycine Gel (XP04202BOX, Invitrogen) along with PageRuler Plus Prestained Protein Ladder (#26619, Thermo Scientific) in MOPS Running Buffer (BF-1131-1000, Baltimore Bioworks) at 120 VDC. The gel was transferred onto PVDF membrane at 4 °C at 30 VDC overnight. 5% nonfat dry milk dissolved in PBS with 0.01% Tween 20 used as the blocking reagent. Blots were incubated in 1:1000 NCAM-L1 antibody (UJ127.11, sc-53386, #B2117, Santa Cruz Biotechnology) or 1:1000 GAPDH antibody (0411, sc-47724, #D0419, Santa Cruz Biotechnology) with 1:1000 TSG 101 antibody (4A10, GTX70255, GeneTex) in 1% nonfat dry milk in PBS/Tween 20 for 1 h at room temperature. The blots were washed three times with PBS/Tween 20 for 10 min each and incubated with 1:50,000 HRP-conjugated secondary antibody (#115-035-062, Jackson ImmunoResearch) for 1 h. Finally, the blots were washed three times with PBS/Tween 20 and incubated with the chemiluminescent substrate (Lumigen ECL Ultra, TMA-6) for 2 min. The blots were imaged using BioRad ChemiDoc Imaging System.

### 4.4. Exosome Uptake

To determine exosome binding and uptake rates of cultured glioma cells, exosomes were labeled with fluorescent membrane dye DiO and were added to 50% confluent 10 cm plates of T98G/shL1 cells. Labeling of exosomes was done by adding 1 μL of Vybrant DiO (Invitrogen Molecular Probes cat# V22886) to 10 μL of exosomes for 30 min, followed by rinsing with PBS. T98G/shL1 cells in a 10 cm dish were incubated for 1–9 h with the labeled exosomes at 37 °C and then rinsed with PBS. Adherent cells were lightly trypsinized 0.05% trypsin/0.02% EDTA to suspend them, and cells were analyzed by flow cytometry to determine fluorescence of the cells using a Becton Dickinson FACSCalibur with 488 nm excitation and detection using the FL1 channel.

### 4.5. SuperScratch Assay

This method [30] was used to evaluate the motility rates of cultured cells after a “wound” was made in confluent cell monolayers in 6-well plate wells (35 mm diameter). T98G/shL1 or U-118 cells were grown in complete media until about 75% confluency. A 1000 µL pipette tip was used to make a straight scratch through the cultured monolayer. Scratched monolayers were rinsed with PBS to remove debris and low serum media was added. Next, any additional treatments or exosomes were added to the plate. For *SuperScratch* cell motility and cell cycle assays, 35 mm wells of cells were treated with either 3 mL of straight supernatant fraction or 5 μL of an exosome preparation diluted in 3 mL of fresh low serum media. Lids of 6-well plates were sealed with petroleum jelly on 3 sides to minimize evaporation and placed in the environmental chamber on our time-lapse microscope system built on a Nikon TE2000-E inverted microscope [30]. MetaMorph software (Molecular Dynamics; ver.7.6.0.0) was used to control the microscope system and return to specific locations along the scratch edge to acquire images approximately every 10 min for a 24 h period using a Nikon 20× ELWD Plan Fluor phase contrast objective. After images were acquired, 15–20 cells per experimental condition were tracked from the collected images to determine the migration rate during 24 h cycle using the Track Points analysis function in MetaMorph. The average migration was calculated and used for migration rate as before [12,13,14]. For example, when cells are tracked every 10 min over 20–24 h, this results in thousands of individual velocity values for each scratch edge (field of view), and each condition (e.g., U-118 MG cells + T98G exosomes) was analyzed in 2 or 3 fields of view in a single culture well. Then, data from the same conditions from several independent experiments were pooled to get the bar graph values of “overall average velocity,” and each bar may represent 6000–8000 or more averaged velocity values from several different fields of view and several different experiments.

### 4.6. Exosome Microscopy

To visualize exosome binding to cells, exosomes stained with lipophilic dyes FM 4–64 (Life technologies Molecular Probes cat# T-13320) or Vybrant DiO (Molecular Probes), or unstained exosomes, were added to T98G/shL1 cells in culture. To do this, each dye was added to the exosome fraction and incubated for 30 min at 37 °C. Excess dye was removed by washing exosomes with PBS twice and centrifugation to re-pellet exosomes. Live cells on coverslips were incubated with the stained exosomes for approximately 60 min at room temperature. Cells then were rinsed and fixed with 1% paraformaldehyde in PBS. Fixed cells incubated with attached exosomes were incubated with primary antibody against L1 (UJ127; cat# sc-53386, Santa Cruz Biotechnologies) for 1 h at room temperature and rinsed three times. Next, they were incubated with Alexa 488-conjugated secondary goat anti-mouse antibody (Jackson ImmunoResearch). Each preparation was stained briefly with 10 ug/mL bisbenzamide in PBS for nuclear staining. Coverslips were mounted on slides in a glycerol-based mounting medium, visualized, and photographed using a Nikon Plan Apo 60x oil immersion objective (N.A., 1.4) using the appropriate filter cube on a Nikon Microphot FX microscope and DXM1200 color CCD camera.

### 4.7. DNA Content/Cell Cycle and Cell Count Analyses

To evaluate the effects of the treatments on advancement through the cell cycle, each cell line was analyzed for DNA content using flow cytometry. Non-confluent (25%) cell cultures were trypsinized and transferred to a 15 mL tube in 2 mL of Soybean Trysin Inhibitor/DNase I. These tubes were centrifuged at 800 rpm and cell pellets were resuspended in a mixture of 500 µL PBS and 4.5 mL cold 70% ethanol and placed at −20 °C. After 4-7 days, the cells were pelleted, rinsed in PBS, and resuspended in 1 mL of DNA staining solution (200 µg/mL of DNase-free RNase A and 20 µg/mL propidium iodide in PBS). After the cells were incubated for 30 min at room temperature in the dark, they were analyzed for DNA fluorescence on a FACSCalibur flow cytometer using the FL2 channel. The percentage of cells in different cell cycle stages was determined from FL2-A data using ModFit *LT* software (Verity Software House; ver. 3.1). The results shown are from individual experiments. To supplement the FACS cell cycle analysis, cells were plated at 10% confluency and grown overnight. The next day, 10 spots were marked using an objective marker and counted for initial cell numbers within the circles. Next, the plates were washed twice with PBS. The appropriate treatment in low serum media was added to the plates and allowed to remain on the cells until the next day. 24 h later, the cells in the 10 marked circles were counted again. A cell count ratio was calculated by dividing the final counts by the initial counts. These values were graphed and used to determine any increases in cell division based upon treatment group. Cell cycle and cell count analyses were performed once.

### 4.8. FGFR and FAK Inhibition

To inhibit FGFR activity, cultures were treated with 50 nM PD173074 (Tocris), which is selective for FGFRs at this concentration (IC_50_ values are 5 and 21.5 nM for FGFR3 and FGFR1, respectively; Tocris website). To inhibit FAK activity, cultures were treated with 10 nM PF 431396 (Tocris), which also inhibits proline-rich tyrosine kinase 2 (PYK2) (IC_50_ values are 2 and 11 nM respectively; Tocris website).

### 4.9. TEM Imaging

To further characterize isolated exosomes, exosomes were resuspended in PBS and placed on 400 mesh formvar/carbon coated copper grids, washed with water and negatively stained with 2% uranyl acetate. Samples were imaged on a Zeiss Libra 120 transmission electron microscope operating at 120 kV, and images were acquired with a Gatan Ultrascan 1000 CCD camera.

### 4.10. In Vivo Invasion Analysis

The in vivo system used in this work was the White Leghorn chicken embryo. Fertilized eggs were obtained from the University of Delaware Department of Animal and Food Sciences. These embryos were incubated at 37.5 °C to embryonic day (E) 5. On E5, a window was cut into the egg and the air space membrane was removed. Approximately 10 µL of GBM cell suspension (~10^5^ cells) was microinjected into the chick optic tectum using a PV830 pneumatic picopump (World Precision Instruments; Sarasota, FL). The GBM cell suspension contained cells, exosomes, low serum media, Fast Green FCF dye, and 30% Matrigel. For injections, 50 µL of T98G/shL1 or /pLKO.1 exosomes were added to the cell prep for injections. The total volume of injection sample was 430 µL (129 µl Matrigel + 50 µL shL1or pLKO.1 exosomes + 251 µL low serum DMEM). The total cell count was 4.3 × 10^6^ cells in an injection sample (injection conc. is 10^7^ cells/mL). After injection, 3 to 4 drops of sterile ampicillin solution (40 mg/mL) were added over the embryo. Transparent tape was placed over the window and the embryo was incubated until E9.

On E9, the embryos were sacrificed and dissected to obtain whole brains. These brains were fixed in 2% paraformaldehyde in PBS for 24–48 h. After fixation, the brains were embedded in embedding media composed of 3.5% agar and 8% sucrose in PBS. Next, the brains were sectioned at 300 microns using a Vibratome Series 1000 Sectioning System (Ted Pella, Redding, CA) for microscopic observation. These sections were mounted on slides in a buffered glycerol mounting media and sealed with nail polish. They were stored at in the dark before visualization using a Nikon Microphot FX epifluorescence microscope equipped with a Nikon DXM1200 color CCD camera. Tumors were scored as “attached” if there were continuous cells from the labeled tumor to the brain wall, which sometimes did not occur (i.e., some tumors grew in the ventricle unattached to the brain wall). Attached tumors were scored as “invasive” if fluorescent tumor cells visibly advanced beyond the generally smooth tumor margin of attachment into the surrounding chick brain tissue as single cells or groups of cells as before [12]. Vibratome sections also were observed on a Nikon E800 epifluorescence microscope equipped with a Nikon C1 confocal scan head and 488 nm argon and 633 nm HeNe lasers, and confocal images were obtained using a 10× Plan Apo objective.

### 4.11. Statistical Analysis

To evaluate the data collected, all data were analyzed by Student’s T-test or ANOVA. After data was averaged for comparison of each treatment, these treatment groups averages were used to calculate standard deviation and standard error. These means were used in ANOVA to see if there was a difference in the treatment groups. The T-test was used to determine the significance of two samples that were compared when ANOVA found significance of treatment groups. All data analysis was performed in Microsoft Excel.

## 5. Conclusions

Our data show that minute L1-decorated exosomal vesicles are released by GBM cells, and they stimulate GBM cell motility, proliferation, and invasiveness, L1-decorated exosomes significantly increased cell velocity in three human glioma cells tested (T98G/shL1, U-118 MG, and primary GBM cells) in a highly quantitative motility assay compared to L1-reduced exosomes from L1-attenuated T98G/shL1 cells. They also caused a marked increase in cell proliferation as determined by DNA cell cycle analysis and cell counting. In addition, L1-decorated exosomes facilitated initial GBM cell invasion when mixed with non-invasive T98G/shL1 cells in our chick embryo brain tumor model.

## Figures and Tables

**Table 1:** Cell motility rates with exosome vs. supernatant treatment.

Glioma Cells	Treatment	Velocity μm/min. (± s.e.m.)
U-118 MG	Plain cells	0.19 (0.015)
	T98G Exosomes	0.34 (0.017)
	T98G Supernatant	0.19 (0.006)
T98G/shL1	Plain cells	0.16 (0.007)
	T98G Exosomes	0.23 (0.013)
	T98G Supernatant	0.17 (0.005)
T98G/dFGFR	Plain cells	0.09 (0.005)
	T98G Exosomes	0.11 (0.008)
	T98G Supernatant	0.04 (0.003)

**Table 2:** Proliferation rates with exosomes.

Glioma Cell Type	Treatment	% Cells in S Phase	Cell Count Ratio
U-118 MG	T98G exosomes	35.4	2.3
	Untreated Cells	23.1	1.3
T98G/shL1	T98G exosomes	43.9	1.6
	Untreated Cells	34.7	1.1
T98G/dFGFR	T98G exosomes	44.3	N.D.
	Untreated Cells	35.2	N.D.

**Table 3:** In vivo tumors and invasion.

Cell Type	Exosome Type	L1 Status		Embryos Analyzed	Attached Tumors	Brains with Invasion	Percent Invasion
		Cells	Exosomes				
T98G/pLKO.1	None	+	None	24	20	18	90%
T98G/pLKO.1	T98G	+	+	18	17	17	100%
T98G/pLKO.1	T98G/pLKO.1	+	+	24	19	18	95%
T98G/pLKO.1	T98G/shL1	+	-	16	14	12	86%
T98G/shL1	None	-	None	24	23	0	0%
T98G/shL1	T98G	-	+	18	17	16	94%
T98G/shL1	T98G/pLKO.1	-	+	16	14	10	71%
T98G/shL1	T98G/shL1	-	-	24	18	0	0%

## Abbreviations

FAK	focal adhesion kinase
FGFR	fibroblast growth factor receptor
GBM	glioblastoma
L1CAM	L1 cell adhesion molecule
